# Anterior Glenohumeral Instability: Classification of Pathologies of Anteroinferior Labroligamentous Structures Using MR Arthrography

**DOI:** 10.1155/2013/473194

**Published:** 2013-10-02

**Authors:** Serhat Mutlu, Mahir Mahıroğullari, Olcay Güler, Bekir Yavuz Uçar, Harun Mutlu, Güner Sönmez, Hakan Mutlu

**Affiliations:** ^1^Kanuni Sultan Suleyman Education and Research Hospital, Department of Orthopaedic Surgery, Istanbul, Turkey; ^2^Istanbul Medipol University, Istanbul, Turkey; ^3^Nisa Hospital, Istanbul, Turkey; ^4^Dicle University, Diyarbakır, Turkey; ^5^Taksim Education and Research Hospital, Istanbul, Turkey; ^6^Gülhane Military Medical Academy Education Hospital, Istanbul, Turkey

## Abstract

We examined labroligamentous structures in unstable anteroinferior glenohumeral joints using MR arthrography (MRA) to demonstrate that not all instabilities are Bankart lesions. We aimed to show that other surgical protocols besides classic Bankart repair are appropriate for labroligamentous lesions. The study included 35 patients (33 males and 2 females; mean age: 30.2; range: 18 to 57 years). MRA was performed in all patients. The lesions underlying patients' instability such as Bankart, anterior labral periosteal sleeve avulsion (ALPSA), and Perthes lesions were diagnosed by two radiologists. MRA yielded 16 diagnoses of Bankart lesions, 5 of ALPSA lesions, and 14 of Perthes lesions. Albeit invasive, MRA seems to be a more reliable and accurate diagnostic imaging modality for the classification and treatment of instabilities compared to standard MRI.

## 1. Introduction

Anterior shoulder instability is one of the most common orthopedic problems. Glenohumeral (GH) instability occurs mostly in young, active males and is usually caused by traumatic injury. Recurrent subluxations and dislocations of the GH joint occur as a result of changes in bone, cartilage, and soft tissues and are referred to as habitual dislocation of the shoulder [1, 2]. This problem, which causes severe morbidity that interferes with daily and sporting activities, is associated with many bone and soft tissue changes. The glenohumeral ligament, its inferior part in particular, is one of the most important passive stabilizers of the shoulder. The glenoid labrum contributes to stability of the shoulder by deepening the glenoid fossa with ligamentous attachments [3, 4]. A number of anterior instabilities can be associated with glenohumeral ligament lesions and with lesions of complex ligamentous structures that the glenohumeral ligament forms with neighboring tissues. The main instabilities are Bankart lesion (avulsion of the anterior glenoid labrum from the bone), glenoid edge fracture, Hill-Sachs lesion (osseous defect due to dislocation of the posterosuperior lateral humeral head), and loose body [1]. In addition, the Perthes lesion, a labroligamentous avulsion in which the scapular periosteum remains intact, and the ALPSA lesion, a medial displacement of the anteroinferior labral ligamentous complex with an intact scapular periosteum, should be considered potential causes of shoulder instability [5, 6].

The identification of anterior labral avulsion, capsular laxity, and other pathologies of the GH joint is of great significance in treatment planning. The methods used to identify these pathologic changes include shoulder radiography with different angles of view as well as computerized tomography (CT), CT arthrography (CTA), magnetic resonance (MR), and magnetic resonance arthrography (MRA) [7–11]. Osseous changes such as lesions of the glenoid and humeral head can be imaged by CT, whereas soft tissue changes such as labral and ligamentous avulsion and capsular laxity can be identified most clearly by MR. Nonetheless, the utility of MRA for precise localization of glenohumeral lesions and identification of the relationships between bone and soft tissue is beyond doubt [12, 13]. 

The purpose of this study was to show that not all instabilities result from Bankart lesions by in-depth evaluation of bone and soft tissue changes in unstable anterior-inferior glenohumeral joints by MRA and to draw attention to other lesions causing instabilities and to the various treatment approaches.

## 2. Patients and Methods

After standard MRI, MRA was performed in 35 shoulders of 35 patients with recurrent shoulder dislocation (at least three dislocations) who were diagnosed clinically with GH instability between June 2006 and October 2012. Thirty-three patients were males and two were females (mean age: 30.2; range: 18–57 years). These patients were selected from 47 patients with chronic shoulder instabilities. Twelve of forty-seven patients were diagnosed with Bankart lesion based on MR imaging results, whereas pathology involving labroligamentous structures could not be diagnosed with certainty in the 35 patients included in the study. MRA images from the 35 patients were evaluated independently by two radiologists.

This study was planned as a retrospective patient file analysis with the permission of the ethics committee. Before MRA, contrast material was injected via the posterior arthroscopic portal by an orthopedist under the guidance of radiologist-operated ultrasound. Injections were performed following topical anesthesia (prilocaine hydrochloride 2% Citanest) while patients were in a semiprone position with the arm held flexed perpendicular to the body for the duration of imaging. The ultrasound probe was placed parallel to the humerus, along the same axis as the infraspinatus in the posterior direction, and angled to visualize the humeral head and posterior glenoid rim. Under the guidance of ultrasound, a 20-gauge spinal needle was inserted through fatty planes and the infraspinatus muscle and advanced between the humeral head and posterior glenoid rim through the capsule. After the capsule was entered, ~12–16 mL of gadolinium diluted 4/1000 in isotonic solution was administered. Distension of the capsule was evaluated by ultrasound. The process took ~10 min. After the intra-articular injection, the shoulder joint was moved to allow the contrast material to spread inside the capsule. 

Ten to fifteen minutes after administration of contrast agent, MRA was performed using a 1.5 Tesla imaging scanner. While patients were in a supine position with the arm externally rotated (for effective evaluation of the rotator cuff and glenohumeral ligaments), selective fat-suppressed axial, coronal, and sagittal T1A-weighted (repetition time: 713 ms, echo time: 12 ms) images were obtained using a shoulder coil. The image matrix was 256 × 256 pixels, section thickness was 4 mm, the gap was 1 mm, and the field of view was 220 mm. 

## 3. Results

**Figure 1:**
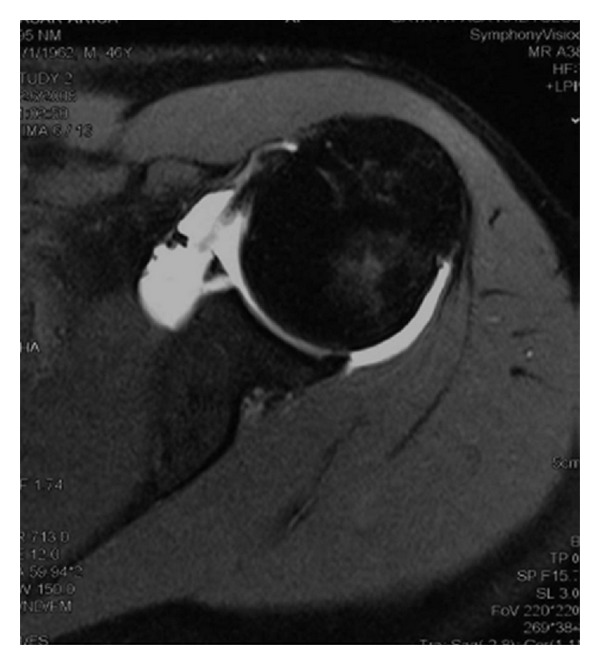
Bankart lesion.

**Figure 2:**
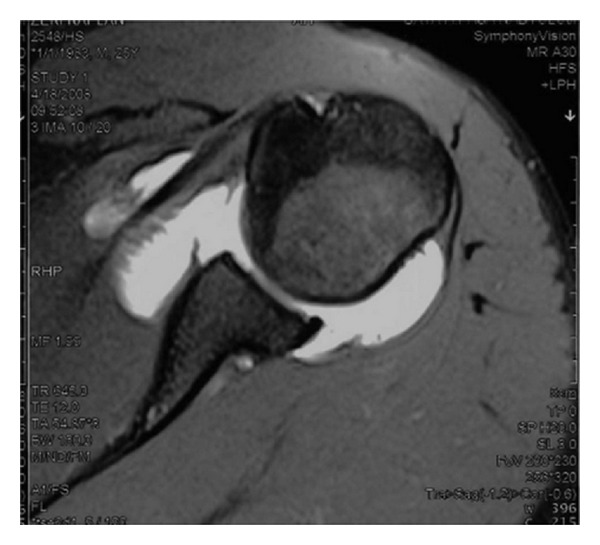
ALPSA lesion.

**Figure 3:**
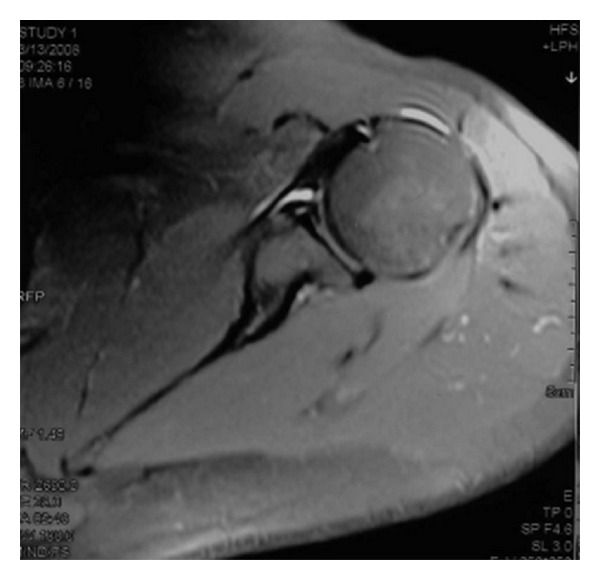
Perthes lesion.

MRA images revealed capsular distension due to the injection of radioopaque contrast material. Based on the results of MRA, 16 patients were diagnosed with Bankart lesions (Figure 1), 5 with ALPSA lesions (Figure 2), and 14 with Perthes lesions (Figure 3). Twenty-nine patients also had Hill-Sachs lesions due to shoulder dislocation in the posterior surface of the corpus humeri.

MRA images were interpreted and reported separately by two radiologists; their diagnoses agreed completely (100%). The diagnoses were categorized and the results are presented as percentages (Table 1).

## 4. Discussion

The glenohumeral joint has the largest range of motion of all joints in the human body. The joint capsule, ligaments, and muscles help to resolve the incongruity between the humeral head and the glenoid cavity in the glenohumeral joint. Therefore, the shoulder joint is prone to recurrent dislocation [1, 14, 15]. The most common shoulder dislocation is traumatic anterior unidirectional glenohumeral instability, which occurs most often after acute anterior shoulder dislocation [2, 14]. The identification of labroligamentous and capsular pathologies by radiological methods after the acquisition of patient history and a detailed physical examination is of great importance in treatment planning. GH instability after acute dislocation is more common among young adults [2, 7, 14]. Conventional radiographic and CT examinations of the shoulder, though inefficient for the evaluation of soft tissues, are important for the evaluation of bone structures in shoulder dislocations [7, 16].

A prospective analysis of 121 MR arthrograms by Palmer and Caslowitz reported that MRA detects labral abnormalities with a sensitivity of 92% and a specificity of 92%. In another study, Chandnani et al. [17] compared MR and CT arthrography with MRA in the same patient group and reported that MRA was superior.

Because the superiority of MR imaging in the detection of soft tissue lesions of the shoulder is beyond doubt, it is the most widely used diagnostic method before treatment. We propose that MRA is superior to MR imaging because it detects lesions that can be missed by MR imaging, through the expansion of the capsule by the contrast material, which reveals ligament cleavage. MRA can therefore better guide selection of the optimum treatment approach.

Gartsman divided anterior labrum tears into three types: Type A, in which the labrum is separated from the glenoid bone but remains at the level of the glenoid joint, Type B, in which the labrum is separated from the glenoid bone and retracted medially, and Type C, in which the labrum is separated from the glenoid bone, retracted, and has healed medially on the glenoid, equivalent to an ALPSA lesion. Gartsman also reported that surgical dissection should be performed before repair and the labrum should be mobilized to the superolateral region in Type B and Type C lesions [18]. In this study, as also reported by Gartsman, we attempted to underline the fact that not all anterior-inferior labroligamentous lesions are Bankart lesions; that is, some might be other Bankart-like lesions. Even though these lesions can be diagnosed by imaging during surgery, preoperative identification of these lesions helps surgeons plan the intervention, guiding the choice of which lesion to target.

Because MRA is associated with swelling and tightening of the joint, it is highly effective in the identification of labroligamentous structures. Even though, unlike standard MRA, MRA of the shoulder is invasive, it is considerably useful in the classification of lesions. Preoperative identification of labroligamentous structures is of great importance because accurate diagnosis is required to identify the appropriate surgical procedure, which may not be Bankart repair as would be assumed from noninvasive MR imaging. Provencher reported that ALPSA-type lesions are associated with twofold higher bone loss during surgical procedures compared to anterior instability alone [19].

Habermeyer et al. suggested that the evolution of ALPSA lesions depends on time and recurrence and that ALPSA lesions do not usually occur with first-time dislocations [20]. In the study presented here, all patients experienced three or more recurrences of dislocation.

MRI with intra-articular contrast material is more specific and sensitive than other imaging methods in the identification of Bankart and more complex lesions (Perthes and ALPSA). The importance of preoperative identification of lesions in reducing postoperative recurrence risk is evident. The failure rate after surgical repair has been reported to be twice as high in ALPSA lesions compared to that in Bankart repair [21]. Habermeyer et al. reported that the ALPSA lesion is chronic by nature [20], which, along with increased bone loss during surgery, likely underlies increased posttreatment failure [22]. Thus, MRA patients diagnosed with ALPSA and their relatives should be informed of the risk of failure prior to acquiring informed consent for surgical procedures.

ALPSA lesions are more challenging than other lesions to visualize arthroscopically from the standard posterior portal. Since the labroligamentous complex has healed medially, it cannot be visualized from the point where the labral tissue is separated. These lesions are more accurately diagnosed by visualization from the anterosuperior portal. Upon imaging from this view, ALPSA lesions would be repaired by elevating the labroligamentous complex of the scapular neck and bringing it back to the anatomic position on the glenoid face [23].

Perthes lesions, also referred to as partial labral detachments, can be missed on routine MRI and even on arthroscopy because they can heal spontaneously through the synovial membrane, granulation tissues, and periosteal fibers. Thus, there may be no clinical findings despite symptoms of shoulder instability, in which case MRA is superior for diagnosis and guiding treatment because it can visualize partial tears revealed by contrast material-induced tension within the capsule [24].

The number of cases in this study was limited, so the distribution and variety of lesions may not be representative of a larger population. However, even in this small series, there was a wide variety, supporting our initial hypothesis that many types of lesions underlie shoulder joint instability. Another limitation of this study is that MRA diagnoses were not confirmed surgically.

In conclusion, MRA is an efficient technique for the diagnosis and classification of anteroinferior labroligamentous lesions in shoulder joint instability. Although degenerative changes and fibrous tissues make the diagnosis of chronic cases difficult, MRA is beneficial before planning arthroscopic surgical intervention.

## Figures and Tables

**Table 1:** Distribution of lesions underlying shoulder instability, as diagnosed by MRA.

Bankart lesion	16 (45.72%)
Perthes lesion	14 (40%)
ALPSA lesion	5 (14.28%)

## References

[1] Liu SH, Henry MH (1996). Anterior shoulder instability: current review. *Clinical Orthopaedics and Related Research*.

[2] Matsen FA, Thomas SC, Rockwood CA, Wirth MA, Rockwood CA, Matsen FA (1998). Glenohumeral instability. *The Shoulder*.

[3] O’Connell PW, Nuber GW, Mileski RA, Lautenschlager E (1990). The contribution of the glenohumeral ligaments to anterior stability of the shoulder joint. *American Journal of Sports Medicine*.

[4] Ovesen J, Nielsen S (1985). Stability of the shoulder joint: cadaver study of stabilizing structures. *Acta Orthopaedica Scandinavica*.

[5] Neviaser TJ (1993). The anterior labroligamentous periosteal sleeve avulsion lesion: a cause of anterior instability of the shoulder. *Arthroscopy*.

[6] Neviaser TJ (1993). The GLAD lesion: another cause of anterior shoulder pain. *Arthroscopy*.

[7] Blum A, Coudane H, Molé D (2000). Gleno-humeral instabilities. *European Radiology*.

[8] Rafii M, Firooznia H, Bonamo JJ (1987). Athlete shoulder injuries: CT arthrographic findings. *Radiology*.

[9] Rafii M, Minkoff J (1998). Advanced arthrography of the shoulder with CT and MR imaging. *Radiologic Clinics of North America*.

[10] Roger B, Skaf A, Hooper AW, Lektrakul N, Yeh L, Resnick D (1999). Imaging findings in the dominant shoulder of throwing athletes: comparison of radiography, arthrography, CT arthrography, and MR arthrography with arthroscopic correlation. *American Journal of Roentgenology*.

[11] Stevens KJ, Preston BJ, Wallace WA, Kerslake RW (1999). CT imaging and three-dimensional reconstructions of shoulders with anterior glenohumeral instability. *Clinical Anatomy*.

[12] Palmer WE, Caslowitz PL (1995). Anterior shoulder instability: diagnostic criteria determined from prospective analysis of 121 MR arthrograms. *Radiology*.

[13] Waldt S, Burkart A, Imhoff AB, Bruegel M, Rummeny EJ, Woertler K (2005). Anterior shoulder instability: accuracy of MR arthrography in the classification of anteroinferior labroligamentous injuries. *Radiology*.

[14] Rowe CR, Zarins B, Ciullo JV (1984). Recurrent anterior dislocation of the shoulder after surgical repair: apparent causes of failure and treatment. *Journal of Bone and Joint Surgery A*.

[15] Turkel SJ, Panio MW, Marshall JL, Girgis FG (1981). Stabilizing mechanisms preventing anterior dislocation of the glenohumeral joint. *Journal of Bone and Joint Surgery A*.

[16] Pancione L, Gatti G, Mecozzi B (1997). Diagnosis of Hill-Sachs lesion of the shoulder: comparison between ultrasonography and arthro-CT. *Acta Radiologica*.

[17] Chandnani VP, Yeager TD, DeBerardino T (1993). Glenoid labral tears: prospective evaluation with MR imaging, MR arthrography, and CT arthrography. *American Journal of Roentgenology*.

[18] Gary M, Gartsman MD (2008). *Shoulder Arthroscopy*.

[19] Provencher MT Glenoid bone loss in patients with instability: the significance of an ALPSA lesion.

[20] Habermeyer P, Gleyze P, Rickert M (1999). Evolution of lesions of the labrum-ligament complex in posttraumatic anterior shoulder instability: a prospective study. *Journal of Shoulder and Elbow Surgery*.

[21] Ozbaydar M, Elhassan B, Diller D, Massimini D, Higgins LD, Warner JJP (2008). Results of arthroscopic capsulolabral repair: bankart lesion versus anterior labroligamentous periosteal sleeve avulsion lesion. *Arthroscopy*.

[22] Bernhardson A Glenoid bone loss in patients with shoulder instability: the significance of the ALPSA(anterior labroligamentous periosteal sleeve avulsion) lesion.

[23] Provencher MT, Romeo AA (2010). *Shoulder Instability, A Comprehensive Approach*.

[24] Provencher MT, Romeo AA (2010). *Shoulder Instability, A Comprehensive Approach*.

